# Palladium-Catalyzed Arylations towards 3,6-Diaryl-1,3a,6a-triazapentalenes and Evaluation of Their Fluorescence Properties

**DOI:** 10.3390/molecules29102229

**Published:** 2024-05-09

**Authors:** Yingchun Wang, Tomas Opsomer, Flip de Jong, Davy Verhaeghe, Maarten Mulier, Luc Van Meervelt, Mark Van der Auweraer, Wim Dehaen

**Affiliations:** 1 Sustainable Chemistry for Metals and Molecules, Department of Chemistry, KU Leuven, Celestijnenlaan 200F, 3001 Leuven, Belgium; 2 Molecular Imaging and Photonics, Department of Chemistry, KU Leuven, Celestijnenlaan 200F, 3001 Leuven, Belgium; flip.dejong@kuleuven.be (F.d.J.); mark.vanderauweraer@kuleuven.be (M.V.d.A.); 3 Biochemistry, Molecular and Structural Biology, Department of Chemistry, KU Leuven, Celestijnenlaan 200F, 3001 Leuven, Belgium; luc.vanmeervelt@kuleuven.be

**Keywords:** triazapentalene, 1,3a,6a-triazapentalene, CH arylation, Suzuki coupling, photophysical properties, fluorescence

## Abstract

Methyl 4-(1,3a,6a-triazapentalen-3-yl)benzoate (**TAP1**) shows interesting properties as a small molecule fluorophore. In the search for post-functionalization methods, palladium-catalyzed arylation reactions were demonstrated. Direct CH arylation reactions of **TAP1** with various aryl halides resulted in 3,6-diaryltriazapentalenes **TAP4**, although mostly in poor yields. Bromination of **TAP1** followed by Suzuki coupling, on the other hand, requires a more delicate procedure, but gave arylated products with the same regiochemistry (**TAP4**) in moderate to good yields. The structure of 6-phenyltriazapentalene **TAP4a** was confirmed by crystallographic analysis. In addition, the effect of the C6 arylation on the fluorescent properties of 3-aryl-1,3a,6a-triazapentalenes was studied in dichloromethane at room temperature and in 2-methyltetrahydrofuran at 77 K, while the photophysical properties of two saponified derivatives were measured in acetonitrile.

## 1. Introduction

1,3a,6a-triazapentalene (TAP) has sparked considerable interest as a novel 10π-electron fluorophore due to its small size [1,2,3,4,5,6], and derivatives of it have been used as part of biological imaging probes and sensors [7,8,9,10]. In particular, bicyclic TAPs with various substitution patterns have been explored extensively by the Namba group [4,11,12,13,14], demonstrating their tunable fluorescent properties. Unfortunately, TAPs might suffer from sensitivity to acidic media and/or UV irradiation. The presence of electron withdrawing groups at the C2 or C3 positions, however, was found to increase the stability significantly [3,5]. In 1978, when simple bicyclic TAPs without additional fused rings were reported for the first time by Koga et al., they already emphasized the strong electron-donating character and high reactivity of triazapentalenes, and the stabilizing effect of electron-withdrawing groups [15].

**Scheme 1:**
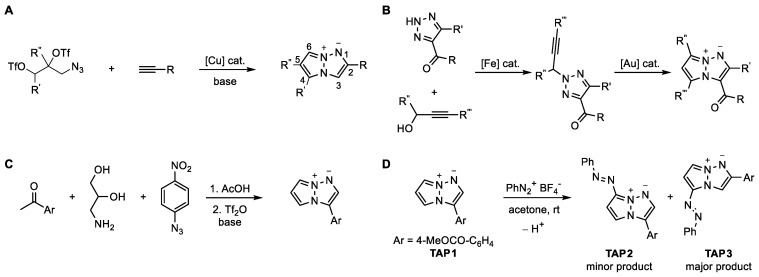
Previously reported synthetic pathways towards TAPs and numbering of the TAP core: (**A**). CuAAC-based synthesis, (**B**). Gold-catalyzed cyclization of propargyl-1,2,3-triazoles, (**C**). Triazolization-based synthesis, (**D**). Azo-coupling of **TAP1**.

Three distinct synthetic pathways to access substituted bicyclic TAP derivatives have been reported. Each one proceeds through the preparation of 1,2,3-triazoles prior to the cyclization reaction that results in the formation of the TAP core. First, Namba et al. demonstrated in a number of reports that the copper-catalyzed azide–alkyne cycloaddition (CuAAC) reaction is a very good starting point. While allowing the incorporation of substituents on each position of the pyrazole moiety, this method generally leads to 3-unsubstituted TAP derivatives (Scheme 1A) [4,11,12,13]. Second, the gold-catalyzed cyclization of 2-propargyl-1,2,3-triazoles, reported by Cai et al., afforded highly functionalized TAPs with electron-withdrawing groups at the C3 position (Scheme 1B) [5]. Complementary to the previous strategies, the triazolization-mediated synthesis provided access to 2-unsubstituted 3-aryl-TAPs from readily available starting materials (Scheme 1C) [3,16]. Interestingly, the resulting compound **TAP1** was found to be highly fluorescent in dichloromethane (DCM), having a quantum yield of fluorescence of 0.79.

In analogy to the reports of the Namba group, derivatization of the 3-amino-1,2-propanediol substrate could be a feasible strategy to acquire structural modifications of **TAP1** on the pyrazole ring. However, in order to avoid multi-step syntheses of starting materials, we opted to study the derivatization of **TAP1** via post-functionalization strategies. Recently, we reported that **TAP1** underwent azo coupling with benzenediazonium tetrafluoroborate in an attempt to introduce a phenyl group via a radical CH arylation. Azo coupling occurred readily at low temperatures, most likely due to the substantial nucleophilic character of triazapentalenes (Scheme 1D) [17]. Surprisingly, the non-fluorescent **TAP3** with the 4-methoxybenzoate group at the C2 position was isolated as the major product and resulted from the ring-degenerate rearrangement of the azo coupled **TAP1**. In addition, attempted reductive cleavages of the azo group lead to the ring opening or cleavage of the TAP core, which further evidenced its instability.

Aiming to expand the variety of substitution patterns of the 3-aryl-TAPs, we herein demonstrate the feasibility of palladium-catalyzed cross-coupling reactions on **TAP1**. This comprises the direct palladium-catalyzed CH arylation with aryl halides, and bromination of **TAP1** followed by Suzuki coupling with arylboronic acids. Suzuki coupling was used earlier to functionalize the pyrazine ring of pyridazino-1,3a,6a-triazapentalenes [18]. To the best of our knowledge, arylation reactions of bicyclic TAPs have not been reported until now.

## 2. Results and Discussion

### 2.1. Synthesis

In an attempt to arylate 1,3a,6a-triazapentalene, a procedure previously reported for the direct CH arylation of BODIPY with aryl halides was applied to **TAP1** [19]. To start our investigation, the reaction was performed with one equivalent of bromobenzene. The expected product **TAP4a** was formed to some extent after 24 h, while a considerable amount of **TAP1** remained unreacted, according to TLC analysis. Aiming to increase the conversion, the reaction mixture was left stirring for another 48 h. Unfortunately, the isolated yield of **TAP4a** was only 21%, and traces of **TAP1** could still be observed. The reaction was then repeated with three equivalents of bromobenzene while keeping the reaction time at 72 h. Favorably, 6-phenyltriazapentalene **TAP4a** was isolated in 57% yield as a major product, accompanied by the diphenylated triazapentalene **TAP5a** in 14% yield (Table 1). The molecular structures of **TAP4a** and **TAP5a** were elucidated by means of single-crystal X-ray diffraction analysis (for **TAP4a**, see Supplementary Materials p. 12) and a 2D-NOESY NMR experiment (for **TAP5a**, see SI Figure S17), respectively. This revealed that bromobenzene had reacted at the C4 and C6 positions, leading to triazapentalenes with unprecedented substitution patterns. As mentioned above, the reaction conditions were adopted from an in-house developed method for the arylation of BODIPY, where arylation occurs at the electrophilic α-positions of the BODIPY core. For this process, a CH cleavage via a concerted metalation–deprotonation in the presence of carbonate as an intramolecular base was proposed as the mechanism [20]. In the case of the electron rich TAP, a palladium-catalyzed electrophilic aromatic substitution mechanism, previously reported for azole compounds, is considered to be more likely [21,22]. The C4 and C6 positions also proved to be the nucleophilic sites in the reaction with benzenediazonium salt [17]. Yet, the first arylation preferentially occurs at the C6 position, while azo coupling mainly occurred at the C4 position prior to rearrangement. In order to achieve a better understanding of the mechanism and to disclose the optimal reaction conditions, further investigations would be required.

As the yield of the reaction with bromobenzene (57%) was already quite satisfying, we decided to evaluate the outcome of the reaction with different bromoarenes rather than further optimizing the reaction parameters. Thus, arylations were tried with various *para*-substituted bromobenzenes (Table 1). 4-bromoalkoxybenzenes (entries 2 and 5) seemed to react faster than other bromobenzenes. In the case of 4-dimethylaminobenzene (entry 4), the reactions proceeded very slowly. Even after three days, no products could be isolated in sufficient amounts for characterization. The experiment with one equivalent of ethyl-4-bromobenzoate gave monoarylated **TAP4c** in 21% yield after 72 h. However, from the reaction with three equivalents of ethyl-4-bromobenzoate, an inseparable mixture of monoarylated **TAP4c** and diarylated **TAP5c** was obtained. Surprisingly, reactions with either one equivalent or three equivalents of 4-bromoanisole only gave monoarylated **TAP4b** in 29% and 40% yield, respectively. It should be noted that the compound **TAP4e** was synthesized in response to the poor solubility of **TAP4b** in organic solvents. As observed during the purification and handling of the arylation products, the stability and solubility of **TAP4c** having an additional electron-withdrawing ester moiety was higher compared to the other examples.

Since the direct CH arylations were not always successful, required long reaction times, and generally resulted in rather poor isolated yields, an alternative approach was investigated, i.e., bromination followed by the Suzuki reaction. Starting from **TAP1**, the bromination reaction was carried out with phenyltrimethylammonium tribromide (PTAB) under basic conditions. The 6-bromotriazapentalene **TAP6a** and 4,6-dibromotriazapentalene **TAP6b** were formed depending on the amount of tribromide that was added (Table 2). Marquet and Jacques [23] reported bromination with PTAB in 1959 and pointed out the high stability and ease of preparation of this reagent. Compared to bromine, PTAB is less electrophilic and reactive toward aromatic rings in THF and could therefore lead to a more controlled functionalization [24]. In the reaction with **TAP1**, the addition of 2.3 equivalents of PTAB in DCM gave rise to the dibrominated **TAP6b** in 85% yield. Monobrominated **TAP6a** was obtained in 77% yield with respect to PTAB when using 6 equivalents of **TAP1**. Because of the light sensitivity of the brominated TAP derivatives, they should be kept in the dark. It should also be mentioned that the 6-bromotriazapentalene **TAP6a** was pure on TLC, while no pure NMR spectrum was obtained due to its poor stability.

The 6-bromotriazapentalene **TAP6a** was directly allowed to react overnight under typical conditions for Suzuki couplings with 1.2 equivalents of boronic acid (Table 3). Arylated triazapentalenes **TAP4** were obtained, and, in general, the yields were high compared to the direct CH arylation reactions (Table 3). Moreover, **TAP4d** could be successfully synthesized via this approach. Unfortunately, under the same conditions, the reaction between 4,6-dibromotriazapentalene **TAP6b** and two equivalents of phenylboronic acid only gave diphenyl **TAP5a** in 28% yield. **TAP4d** did not only exhibit poor solubility in organic solvents, but it also proved to be very unstable under UV irradiation, and therefore, its photophysics were not explored further. In general, the 3,6-disubstituted TAPs could be purified by silica gel column chromatography without significant decomposition. However, the products with electron donating groups are preferentially being protected from light.

**Scheme 2:**
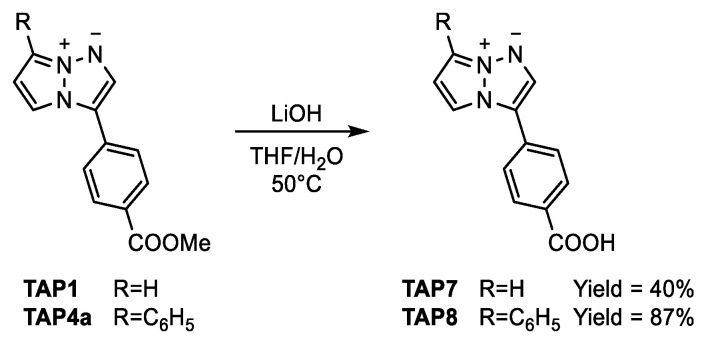
Saponification of **TAP1** and **TAP4a**.

In the framework of our search to functionalize **TAP1**, hydrolysis of the methyl ester was attempted. In the presence of lithium hydroxide, **TAP1** and **TAP4a** could easily be converted to triazapentalenes **TAP7** and **TAP8** bearing a carboxylic acid group amenable to further derivatization (Scheme 2). The saponification of **TAP1** gave a complex reaction mixture after 16 h at 50 °C according to TLC analysis, and a part of **TAP7** decomposed to a deep-green colored product that strongly absorbed on silica. Under the same conditions, compound **TAP8** was obtained in 87% yield.

### 2.2. Photophysical Measurements

**Figure 1:**
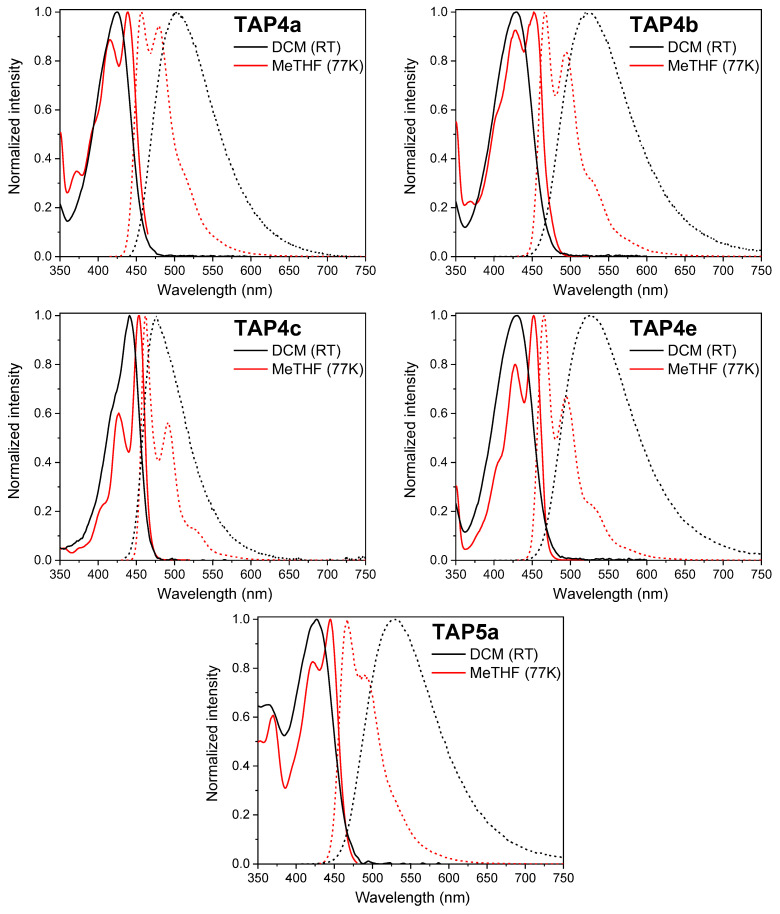
Normalized absorption and emission spectra in DCM at room temperature and normalized excitation and emission spectra in MeTHF at 77 K for **TAP4a**, **TAP4b**, **TAP4c**, **TAP4e**, and **TAP5a**.

**Figure 2:**
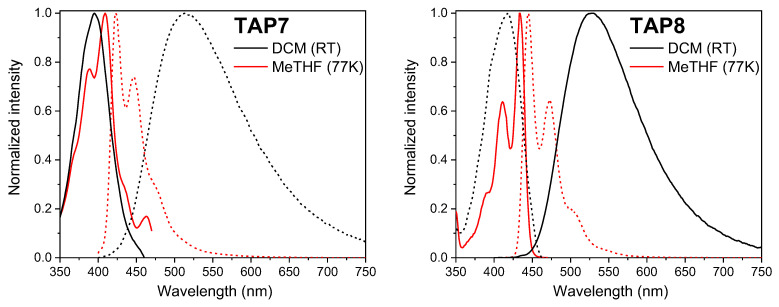
Normalized absorption and emission spectra in ACN at room temperature and normalized excitation and emission spectra in MeTHF at 77 K for **TAP7** and **TAP8**.

Steady-state spectra were taken for **TAP 4a–c**, **4e**, **5a**, **7**, and **8** in DCM at room temperature and in a MeTHF glass at 77K. The spectra for **TAP4** and **TAP5a** are collected in Figure 1, and the spectra for **TAP7** and **TAP8** can be found in Figure 2. All photophysical parameters are listed in Table 4. While at room temperature no clear vibronic progression was observed, at 77 K, the spectra show a pronounced vibrational progression with the 0–0 transitions being the most intense, both in absorption and emission, leading to a nice mirror image between the absorption and emission spectra of all dyes. Compared to **TAP 4a–4b** and **4e**, the 0–1 transition is less intense in **TAP4c**, indicating a smaller change in bond length of the conjugated system upon excitation. This is also reflected in the values of FWHM_em_ which all lie between 2050 and 2330 cm^−1^, except for **TAP4c** (1670 cm^−1^). Compared to **TAP4a**, both absorption and emission spectra are slightly redshifted in **TAP 4b**, **4c**, and **4e**, suggesting a stronger delocalization of the conjugated system over the C6-aryl. In **TAP 4b**, **4c**, and **4e**, the Stokes shift of the 0–0 transitions is smaller compared to **TAP4a**, suggesting a more coplanar C6-aryl. For **TAP8**, a blue shift is observed in MeTHF at 77 K for both absorption and emission, compared to **TAP4a**. This is due to the stronger electron acceptor character of the COOH moiety compared to the COOMe moiety, leading to a weaker stabilization of the LUMO compared to the HOMO, which, based on the resonance forms, will have a large amplitude at C3. For **TAP7**, an additional shoulder was observed around 465 nm in the fluorescence spectrum, which might be due to an impurity. No triplet emission was observed, which either points to internal conversion as a dominant non-radiative decay pathway or efficient intersystem crossing from the triplet state to the ground state.

When comparing the room temperature spectra of **TAP 4a–4e**, **5a**, **7**, and **8** with those at 77 K, one observes in the room temperature spectra a loss of vibrational fine structure, a redshift of the emission maximum over 600 cm^−1^ to 4110 cm^−1^, a blue shift of the absorption maximum over 600 cm^−1^ to 1410 cm^−1^, and a consequent increase in the Stokes shift. It is difficult to assess the extent to which these shifts are due to an increased rotational freedom of the C6-aryl moiety leading to a different degree of coplanarity in the ground and excited state, or to interactions between a permanent electrical dipole of the dye and a polar solvent. The former phenomenon is related to the blocking of internal rotation in aggregates or in the solid state [25]. With the exception of **TAP4c**, the shifts are systematically larger for the emission than for the absorption spectra, indicating in the first case a curvature of the ground state potential energy surface for rotation of the C6-aryl, which is larger than that of the excited state potential energy surface. If, on the other hand, solvent effects play a major role, this means that the excited state dipole moment is larger than that of the ground state. For **TAP4c**, both shifts seem much smaller than those observed for the other compounds. However, one should be careful in drawing conclusions from this observation as, due to the higher relative intensity of the 0–0 transition in **TAP4c** at 77 K, the maximum of the room temperature absorption and emission spectra of **TAP4c** still corresponds to a 0–0 vibronic band, while, due to the broadening of the individual vibronic bands, the position of the maximum of the absorption and emission spectra of **TAP 4a–4b**, **4e**, **5a**, **7**, and **8** resembles more that of the 0–1 vibronic bands.

For the 3,6-diaryl **TAP4a** in DCM, absorption and emission are redshifted compared to **TAP1**, reflecting some degree of conjugation between the **TAP** core and the C6-aryl. This effect is enhanced, especially for the fluorescence, by the addition of a second C4-aryl in **TAP5a**, leading to a larger Stokes shift and FWHM_em_ of **TAP5a**. Analogous to what was observed in MeTHF at 77 K, adding electron donating or accepting substituents to the aryl-C6 yields a further redshift of absorption and emission spectra. This indicates that the effect of the substituents is mainly due to an increase in the size of the conjugated system. Adding electron donating substituents on the 6-phenyl results in larger Stokes shifts (**TAP4b**, **4c**, and **4e**) and FWHM_em_. The effect of an electron withdrawing substituent is less clear as the features of the spectra of **TAP4c** differ from those of the other compounds (cfr. Supra). In contrast to what is observed for the position of absorption and emission maxima, the fluorescence quantum yields follow a clear trend: upon increasing the electron accepting character of the aryl-C6, the fluorescence quantum yields of **TAP4** in DCM drop. As the substituents on aryl-C6 cause no major spectral changes, it is unlikely that the oscillator strength of the S_0_–S_1_ transition will be changed significantly by the substituents; hence, the changes in the fluorescence quantum yields induced by the substituents reflect changes in the rate constant for non-radiative decay. This trend is quite surprising since based on the reorganization energy between the S_0_ and S_1_ state, for which the Stokes shift at 77 K or the FWHM of the emission spectrum are a good indication, one would expect the lowest reorganization energy and the slowest internal conversion for **4c** [26,27,28,29]. Therefore, the trend in the rate constant for internal conversion must reflect the variation of the electronic part of the wave function of the S_1_ state rather than that of the Franck Condon factor. For the saponified **TAP7** (acid form) and **TAP8** (acid form), we were not able to measure the absorption and emission spectra in DCM. However, the absorption spectra in ACN show a small blue shift compared to **TAP1** and **TAP4a** in DCM, while for the emission spectra, a redshift of 1480 cm^−1^ and 760 cm^−1^ is observed for **TAP7** and **TAP8**, respectively, resulting in an increased Stokes shift and FWHM_em_ along with a lower fluorescence quantum yield, which is in line with the observations in a previous publication [3].

## 3. Materials and Methods

### 3.1. Chemicals and Materials

All chemicals were purchased from Acros Organics, Sigma-Aldrich, J&K Scientific, Fluorochem, or TCI Europe, and were used as received. *p*-Nitrophenyl azide [30] and methyl 4-(1,3a,6a-triazapentalen-3-yl)benzoate (**TAP1**) [17] were prepared according to procedures reported in the literature. Dry solvents were purchased from Acros Organics and were used as received. For column chromatography, 70–230 mesh silica gel 60 (Acros) was used as the stationary phase. Spectroscopic solutions were prepared using commercially available solvents of spectroscopic grade.

### 3.2. Instruments

^1^H and ^13^C NMR spectra were recorded on a Bruker Avance 300, Bruker Avance III HD 400, or a Bruker Avance II^+^ 600 spectrometer. Chemical shifts (δ) are reported in parts per million (ppm), referenced to tetramethylsilane (0.00 ppm) as an internal standard for samples in CDCl_3_, or to the solvent signal for samples in DMSO-*d*_6_ (2.50 ppm). ^13^C NMR spectra were referenced to the respective solvent signals (CDCl_3_ 77.16 ppm, CD_3_OD 49.00 ppm and DMSO-*d*_6_ 39.52 ppm). High-resolution mass spectra were acquired on a quadrupole orthogonal acceleration time-of-flight mass spectrometer (Synapt G2 HDMS, Waters, Milford, MA, USA). Samples were infused at 3 µL/min and spectra were obtained in positive ionization mode with a resolution of 15,000 (FWHM) using leucine enkephalin as lock mass. Melting points (not corrected) were determined using a Reichert Thermovar apparatus. Fluorescence spectra were recorded on a HORIBA Jobin Yvon Fluorolog FL3-22 fluorimeter and on an Edinburgh FLS980 fluorimeter. For the measurements at 77 K, the samples were transferred to a clean NMR tube and immersed in an accessory containing a liquid nitrogen dewar. Absolute quantum yields were determined with an integrating sphere and a 0.3% neutral density filter was used when recording the Rayleigh scatter.

### 3.3. Synthesis

#### 3.3.1. Palladium-Catalyzed CH Arylations

To a flame-dried, screw-capped reaction tube equipped with magnetic stirring bar, **TAP1** (50 mg, 1 equiv, 0.21 mmol), K_2_CO_3_ (87 mg, 3 equiv, 0.63 mmol,), Pd(OAc)_2_ (2.5 mg, 5 mol%, 0.011 mmol), PCy_3_HBF_4_ (7.8 mg, 10 mol%, 0.021 mmol), pivalic acid (6.4 mg, 30 mol%, 0.063 mmol), and the bromoarene (1 or 3 equiv) were added and dissolved in dry toluene (2.1 mL). The reaction tube was flushed with nitrogen and the mixture was stirred at 110 °C for the indicated time. Upon completion, the reaction mixture was cooled to room temperature, diluted with EtOAc (10 mL), and washed with water (2 × 10 mL) and brine (1 × 10 mL). The organic layer was subsequently dried over MgSO_4_, filtered, and concentrated under reduced pressure. Further purification was done via column chromatography.

#### 3.3.2. Brominations

**TAP1** was added to an oven-dried round bottom flask equipped with a magnetic stirring bar and dissolved in dry DCM. After adding DIPEA, the reaction mixture was cooled to 0 °C. Next, the phenyltrimethylammonium tribromide (PTAB) was added portionwise while stirring. The mixture was stirred for 2 h at 0 °C. After the mixture was concentrated under reduced pressure at 35 °C, the residue was dissolved in DCM (10 mL) and washed with a saturated Na_2_S_2_O_3_ solution (aq., 1 × 10 mL), NaHCO_3_ (aq., 1 × 10 mL) and water (1 × 10 mL). The organic layer was subsequently dried over MgSO_4_ and concentrated under reduced pressure. Further purification was done via column chromatography using a petroleum ether-DCM-EtOAc gradient (9:1:0–6:3:1) as the eluent.

#### 3.3.3. Palladium-Catalyzed Suzuki Coupling Reactions

To a screw-capped reaction tube equipped with a magnetic stirring bar, 6-bromo-TAP (**TAP6a**, 1 equiv), Pd(PPh_3_)_4_ (5 mol%), and arylboronic acid (1.2 equiv) were added. The mixture was dissolved in THF, after which a solution of K_2_CO_3_ (6 equiv) in water was added with a syringe (for volumes, see Section 3.3.5). Next, the reaction mixture was stirred at 70 °C for 14 h while being covered from the light. The mixture was then cooled to room temperature, diluted with EtOAc (20 mL), and washed with water (2 × 15 mL) and brine (1 × 15 mL). The organic layer was subsequently dried over MgSO_4_, filtered, and concentrated under reduced pressure. Further purification was done via flash column chromatography with pure DCM.

#### 3.3.4. Saponification 

To a stirred solution of **TAP1** or **TAP4a** (24.2 mg, 1 equiv, 0.1 mmol) in 0.7 mL of THF, a solution of LiOH (5 mg, 1.2 equiv, 0,12 mmol) in water (0.3 mL) was added dropwise. After stirring for 16 h at 50 °C, the reaction mixture was cooled to room temperature, and water (10 mL) and 1 M HCl (aq., 1 mL) were added. Next, a yellow precipitate was filtered off, washed three times with ethanol, and dried under vacuum to obtain the pure products.

#### 3.3.5. Experimental Details and Characterization Data

**TAP4a**: (1). Prepared following the general procedure for palladium-catalyzed CH arylations: **TAP1** (50 mg, 1 equiv, 0.21 mmol), bromobenzene (65 mg, 1 equiv, 0.21 mmol) toluene (2.1 mL), 72 h. Purification by column chromatography, using a DCM-EtOAc gradient (100:0–98:2) as the eluent, afforded **TAP4a** (14 mg, 21%) as a yellow solid. (2). Prepared following the general procedure for Suzuki coupling reactions: **TAP6a** (22.4 mg, 1 equiv, 0.07 mmol), Pd(PPh_3_)_4_ (4 mg, 5 mol%, 3.5 µmol), and phenylboronic acid (10.3 mg, 1.2 equiv, 0.084 mmol) in THF (4 mL), K_2_CO_3_ (54 mg, 6 equiv, 0.42 mmol) in water (1 mL). Purification by column chromatography using DCM as the eluent afforded compound **TAP4a** (21.1 mg, 94%) as a yellow solid. Mp: 220–223 °C. ^1^H NMR (400 MHz, CDCl_3_) δ 8.19–8.14 (m, 2H), 8.11 (d, *J* = 8.5 Hz, 2H), 8.02 (d, *J* = 1.2 Hz, 1H), 7.70 (d, *J* = 3.2 Hz, 1H), 7.59 (d, *J* = 8.6 Hz, 2H), 7.52–7.46 (m, 2H), 7.32–7.27 (m, 1H), 7.13 (dd, *J* = 3.2, 1.3 Hz, 1H), 3.94 (s, 3H). ^13^C NMR (101 MHz, CDCl_3_) δ 166.8, 133.6, 132.6, 130.8, 129.0, 128.5, 126.9, 126.6, 124.3, 121.7, 118.3, 112.2, 107.0, 104.2, 52.2. HRMS (ESI-Q-TOF): *m*/*z* [M + H]^+^ calcd for C_19_H_15_N_3_O_2_: 318.1237, found: 318.1234. 

**TAP4b**: (1). Prepared following the general procedure for palladium-catalyzed CH arylations: **TAP1** (50 mg, 1 equiv, 10.21 mmol), 4-bromoanisole (118 mg, 3 equiv, 0.21 mmol), toluene (2.1 mL), 48 h. Purification by column chromatography using DCM as the eluent afforded **TAP4b** (29 mg, 40%) as a yellow solid. (2). Prepared following the general procedure for Suzuki coupling reactions: **TAP6a** (35 mg, 1 equiv, 0.11 mmol), Pd(PPh_3_)_4_ (6.4 mg, 5 mol%, 5.5 µmol), and 4-methoxyphenylboronic acid (20 mg, 1.2 equiv, 0.13 mmol), in THF (4 mL), K_2_CO_3_ (91 mg, 6 equiv, 0.66 mmol) in water (1 mL). Purification by column chromatography using DCM as the eluent afforded compound **TAP4b** (34 mg, 89%) as a yellow solid. Mp: 260–263 °C. ^1^H NMR (300 MHz, DMSO-*d*_6_) δ 8.54 (s, 1H), 8.32 (d, *J* = 3.3 Hz, 1H), 8.21 (d, *J* = 8.9 Hz, 2H), 8.00 (d, *J* = 8.4 Hz, 2H), 7.85 (d, *J* = 8.5 Hz, 2H), 7.50 (d, *J* = 2.0 Hz, 1H), 7.09 (d, *J* = 8.9 Hz, 2H), 3.86 (s, 3H), 3.82 (s, 3H). HRMS (ESI-Q-TOF): *m*/*z* [M] calcd for C_20_H_17_N_3_O_3_: 347.1270, found: 347.1263. 

**TAP4c**: (1). Prepared following the general procedure for palladium-catalyzed CH arylations: **TAP1** (50 mg, 1 equiv, 0.21 mmol), ethyl 4-bromobenzoate (48 mg, 1 equiv, 0. 21 mmol), toluene (2.1 mL), 72 h. Purification by column chromatography, using a DCM-EtOAc gradient (100:0–98:2) as the eluent, afforded compound **TAP4c** (17 mg, 21%) as a yellow solid. (2). Prepared following the general procedure for Suzuki coupling reactions: **TAP6a** (13 mg, 1 equiv, 0.041 mmol), Pd(PPh_3_)_4_ (2.3 mg, 5 mol%, 2.1 µmol), and 4-ethoxycarbonylphenylboronic acid (9.5 mg, 1.2 equiv, 0.049 mmol) in THF (4 mL), K_2_CO_3_ (34 mg, 6 equiv, 0.24 mmol) in water (1 mL). Purification by column chromatography using DCM as the eluent afforded compound **TAP4c** (7.3 mg, 46%) as a yellow solid. Mp: 212–214 °C. ^1^H NMR (300 MHz, CDCl_3_) δ 8.26–8.19 (m, 2H), 8.18–8.08 (m, 4H), 8.02 (s, 1H), 7.70 (d, *J* = 3.0 Hz, 1H), 7.60 (d, *J* = 8.1 Hz, 2H), 7.23–7.18 (m, 1H), 4.40 (q, *J* = 7.2 Hz, 2H), 3.94 (s, 3H), 1.42 (t, *J* = 7.1 Hz, 3H). ^13^C NMR (101 MHz, CDCl_3_) δ 166.7, 166.5, 133.6, 132.5, 132.2, 130.9, 130.4, 128.0, 127.2, 123.5, 122.1, 117.2, 113.0, 108.0, 104.4, 61.1, 52.3, 14.6. HRMS (ESI-Q-TOF): *m*/*z* [M + H]^+^ calcd for C_22_H_19_N_3_O_4_: 390.1448, found: 390.1440. 

**TAP4d:** Prepared following the general procedure for Suzuki coupling reactions: **TAP6a** (10 mg, 1 equiv, 0.03 mmol), Pd(PPh_3_)_4_ (1.7 mg, 5 mol%, 1.5 µmol), and 4-(dimethylamino)phenylboronic acid (6 mg, 1.2 equiv, 0.036 mmol) in THF (4 mL), K_2_CO_3_ (25 mg, 6 equiv, 0.18 mmol) in water (1 mL). Purification by column chromatography using DCM as the eluent afforded compound **TAP4d** (9 mg, 87%) as a deep-yellow solid. Decomposition before melting. ^1^H NMR (300 MHz, DMSO-*d*_6_) δ 8.51 (s, 1H), 8.28 (s, 1H), 8.09 (d, *J* = 8.3 Hz, 2H), 7.99 (d, *J* = 8.2 Hz, 2H), 7.81 (d, *J* = 8.2 Hz, 2H), 7.38 (s, 1H), 6.86 (d, *J* = 8.5 Hz, 2H), 3.86 (s, 3H), 2.97 (s, 6H). HRMS (ESI-Q-TOF): *m*/*z* [M] calcd for C_21_H_20_N_4_O_2_: 360.1586, found: 360.1578. 

**TAP4e**: Prepared following the general procedure for palladium-catalyzed CH arylations: **TAP1** (50 mg, 1 equiv, 0.21 mmol), 1-bromo-4-(hexyloxy)benzene (54 mg, 1 equiv, 0.21 mmol), toluene (2.1 mL), 48 h. Purification by column chromatography, using a DCM-EtOAc gradient (1:0–9:1) as the eluent, afforded compound **TAP4e** (25.2 mg, 29%) as a yellow solid. Mp: 186–188 °C. ^1^H NMR (400 MHz, CDCl_3_) δ 8.15–7.95 (m, 5H), 7.68 (d, *J* = 3.2 Hz, 1H), 7.62–7.53 (m, 2H), 7.09–6.96 (m, 3H), 4.02 (t, *J* = 6.5 Hz, 2H), 3.93 (s, 3H), 1.88–1.73 (m, 2H), 1.63–1.19 (m, 6H), 1.00–0.84 (m, 3H). ^13^C NMR (101 MHz, CDCl_3_) δ 166.9, 158.4, 133.7, 132.8, 130.8, 126.2, 125.9, 121.4, 121.1, 118.6, 115.1, 111.8, 106.2, 104.2, 68.3, 52.2, 31.8, 29.4, 25.9, 22.8, 14.2. HRMS (ESI-Q-TOF): *m*/*z* [M] calcd for C_25_H_27_N_3_O_3_: 417.2052, found: 417.2047. 

**TAP5a**: (1). Prepared following the general procedure for palladium-catalyzed CH arylations: **TAP1** (100 mg, 1 equiv, 0.41 mmol), K_2_CO_3_ (172 mg, 3 equiv, 1.24 mmol), Pd(OAc)_2_ (4,6 mg, 5 mol%, 0.021 mmol), PCy_3_HBF_4_ (15.1 mg, 10 mol%, 0.041 mmol), pivalic acid (13 mg, 30 mol%, 0.13 mmol), bromobenzene (195 mg, 3 equiv, 1.24 mmol), toluene (4.2 mL), 72 h. Purification by column chromatography, using a PE-DCM-EtOAc gradient (6:4:0–6:3:1) as an eluent, afforded the **TAP4a** (76.5 mg, 59%) and **TAP5a** (22.2 mg, 14%) as yellow solids. (2). Prepared following the general procedure for Suzuki coupling reactions: **TAP6b** (40 mg, 1 equiv, 0.10 mmol), Pd(PPh_3_)_4_ (6 mg, 5 mol%, 5 µmol), and phenylboronic acid (30.5 mg, 2.5 equiv, 0.25 mmol) in THF (4 mL), K_2_CO_3_ (86 mg, 6 equiv, 0.6 mmol) in water (1 mL). Purification by column chromatography using DCM as the eluent afforded compound **TAP5a** (11 mg, 28%) as a yellow solid. Mp: 128–130 °C. ^1^H NMR (400 MHz, CDCl_3_) δ 8.24–8.19 (m, 2H), 7.88 (d, *J* = 1.2 Hz, 1H), 7.81 (d, *J* = 8.5 Hz, 2H), 7.55–7.48 (m, 2H), 7.34–7.21 (m, 4H), 7.17 (d, *J* = 1.3 Hz, 1H), 7.14–7.09 (m, 2H), 6.99 (d, *J* = 8.5 Hz, 2H), 3.90 (s, 3H). ^13^C NMR (101 MHz, CDCl_3_) δ 167.0, 135.5, 131.6, 129.2, 129.0, 128.8, 128.6, 128.1, 127.7, 127.7, 127.1, 126.9, 125.8, 124.4, 119.0, 118.8, 113.0, 106.9, 52.2. HRMS (ESI-Q-TOF): *m*/*z* [M + H]^+^ calcd for C_25_H_19_N_3_O_2_: 394.1550, found: 394.1542. 

**TAP6a**: Prepared following the general procedure for bromination: **TAP1** (73 mg, 6 equiv, 0.3 mmol), DIPEA (72 µL, 1.3 equiv, 0.065 mmol), PTAB (19 mg, 1 equiv, 0.05 mmol), DCM (18 mL). Dark green solid (12.3 mg, 77%). **TAP1** (60 mg) was recovered in 82% yield. 

**TAP6b**: Prepared following the general procedure for bromination: **TAP1** (50 mg, 1 equiv, 0.21 mmol), DIPEA (81 µL, 2.2 equiv, 0.462 mmol), PTAB (174 mg, 2.3 equiv, 0.462 mmol), DCM (5 mL). Brown solid (71.4 mg, 85%). ^1^H NMR (300 MHz, CDCl_3_) δ 8.10 (d, *J* = 8.2 Hz, 2H), 7.67 (s, 1H), 7.56 (d, *J* = 8 Hz, 2H), 6.73 (s, 1H), 3.95 (s, 3H). 

**TAP7**: Prepared according to the general procedure for saponification: **TAP1** (24.2 mg, 1 equiv, 0.1 mmol). Yellow solid (9.1 mg, 40%). Mp: 255–257 °C. ^1^H NMR (300 MHz CD_3_OD) δ 8.14–8.05 (m, 3H), 7.95 (d, *J* = 3.0 Hz, 1H), 7.77–7.66 (m, 3H), 6.95–6.88 (m, 1H). ^13^C NMR (101 MHz, CD_3_OD_SPE) δ 166.5, 134.0, 131.6, 129.7, 125.3, 120.2, 110.2, 109.6, 109.5, 104.5. HRMS (ESI-Q-TOF): *m*/*z* [M + H]^+^ calcd for C_12_H_9_N_3_O_2_: 228.0767, found: 228.0760.

**TAP8**: Prepared according to the general procedure for saponification: **TAP4a** (31.7 mg, 1 equiv, 0.1 mmol). Yellow solid (20.8 mg, 87%). Mp: 280–282 °C. ^1^H NMR (400 MHz, DMSO-*d*_6_) δ ^1^H NMR (400 MHz, DMSO-*d*_6_) δ 8.40 (d, *J* = 1.2 Hz, 1H), 8.30–8.23 (m, 3H), 7.98 (d, *J* = 8.4 Hz, 2H), 7.68 (d, *J* = 8.4 Hz, 2H), 7.57 (dd, *J* = 3.3, 1.2 Hz, 1H), 7.50 (t, *J* = 7.8 Hz, 2H), 7.30–7.23 (m, 1H). ^13^C NMR (101 MHz, DMSO) δ 168.4, 138.1, 132.8, 130.0, 128.8, 128.5, 127.5, 126.0, 123.3, 121.2, 115.7, 112.5, 107.5, 104.7. HRMS (ESI-Q-TOF): *m*/*z* [M] calcd for C_18_H_13_N_3_O_2_: 303.1008, found: 303.0999.

### 3.4. X-ray Crystallography

Single crystals of **TAP4a** were obtained by crystallization from toluene. X-ray intensity data were collected at 293(2) K on an Agilent SuperNova diffractometer with Eos CCD detector using MoKα radiation. The images were processed (unit cell determination, intensity data integration, correction for Lorentz and polarization effects, and empirical absorption correction) using CrysAlisPRO [31]. Using Olex2 [32], the structure was solved with the ShelXT [33] structure solution program using Intrinsic Phasing and refined with the ShelXL [34] refinement package using full-matrix least-squares minimization on F^2^. All H atoms were placed in idealized positions and refined in the riding mode. Non-hydrogen atoms were refined anisotropically and hydrogen atoms in the riding mode with isotropic temperature factors fixed at 1.2 times U_eq_ of the parent atoms (1.5 for methyl groups). Crystallographic data for **TAP4a** has been deposited with the Cambridge Crystallographic Data Centre and allocated the deposition number 2151317.

Crystal Data for **TAP4a** (C_19_H_15_N_3_O_2_, M = 317.34 g/mol): orthorhombic, space group *Pna*2_1_, *a* = 6.4743(6), *b* = 7.1508(9), *c* = 33.124(4) Å, *V* = 1533.5(3) Å^3^, *Z* = 4, *T* = 293(2) K, μ(MoKα) = 0.092 mm^−1^, *D_calc_* = 1.374 g/cm^3^, 8290 reflections measured (4.92° ≤ 2θ ≤ 52.74°), 3021 unique (*R*_int_ = 0.0361, *R*_sigma_ = 0.0478) that were used in all calculations. The final *R*_1_ was 0.0580 (I > 2σ(I)), and *wR*_2_ was 0.1440 for all data.

## 4. Conclusions

In summary, novel 3,6-disubstituted-1,3a,6a-triazapentalenes were successfully synthesized by Pd-catalyzed arylation reactions. Yields of the direct CH arylation reactions were not outstanding, but bromination followed by Suzuki coupling was proven to be an effective alternative strategy. Phenyl substituents with an electron donating group had a negative effect on the stability of TAPs, which was previously described by the Namba group for 2-substituted TAPs. Furthermore, poor solubilities in organic solvents were observed after purification for **TAP4b** and **TAP4d** with methoxyphenyl and 4-(dimethylamino)phenyl substituents at the C6 position, respectively. The ethyl benzoate group, on the other hand, had a beneficial effect on both the solubility and stability. In addition, 3,4,6-triaryltriazapentalene **TAP4a** proved to be more stable during saponification than 3,6-diaryltriazapentalenes **TAP1**. Regarding the photophysical properties, fluorescence quantum yields in DCM drop significantly when adding a phenyl group to the C6 position of **TAP1**. Except for the emission of **TAP4c** containing a 4-ethylbenzoate substituent, bathochromic shifts were observed for both the absorption and emission of the arylated TAPs. The measurements in MeTHF glass at 77 K revealed the vibronic progression and showed a nice mirror image of the excitation and emission spectra.

## Figures and Tables

**Table 1:** Synthesis of 3,6-diaryl-1,3a,6a-triazapentalenes via direct CH arylation ^a^.

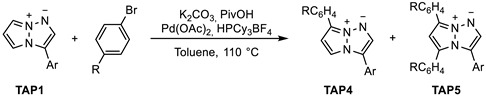						
Entry	Compound	R	Reaction Time	Yield (%) ^b^		
				TAP4	TAP5	TAP1
1	**a**	H	72 h	57 ^e^ (21) ^c^	14 ^e^	- ^d^
2	**b**	OCH_3_	48 h	40 ^e^ (29) ^c^	- ^f^	- ^d^
3	**c**	COOEt	72 h	- ^g^ (21) ^c^	- ^g^	- ^d^
4	**d**	N(CH_3_)_2_	72 h	- ^d^	- ^d^	44
5	**e**	OC_6_H_13_	48 h	- ^e^ (29) ^c^	- ^f^	- ^d^

Ar = 4-MeOCO-C_6_H_4_. ^a^ Experimental conditions: **TAP1** (1 equiv), K_2_CO_3_ (3 equiv), Pd(OAc)_2_ (5 mol%), PCy_3_HBF_4_ (10 mol%), pivalic acid (30 mol%), dry toluene (1 mL per 0.1 mmol of **TAP1**). ^b^ Isolated yields. ^c^ 1 Equiv of aryl bromide. ^d^ Trace or decomposition during reaction. ^e^ 3 Equiv of aryl bromide. ^f^ Not formed. ^g^ Inseparable mixture.

**Table 2:** Bromination of **TAP1**.

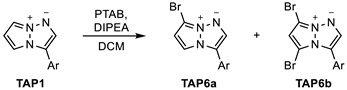			
TAP1 (Equiv)	PTAB (Equiv)	Yield (%)	
		TAP6a	TAP6b
6	1	77	- ^a^
1	2.3	- ^a^	85

Ar = 4-MeOCO-C6H4. ^a^ Not isolated.

**Table 3:** Synthesis of 3,6-diaryl-1,3a,6a-triazapentalenes via Suzuki coupling ^a^.

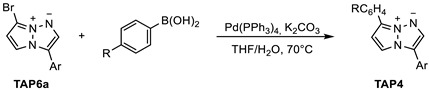			
Entry	Compound	R	Yield (%) ^b^
1	**a**	H	94
2	**b**	OMe	89
3	**c**	COOEt	46
4	**d**	N(CH_3_)_2_	87

Ar = 4-MeOCO-C6H4. ^a^ Experimental conditions: **TAP6a** (0.03–0.11 mmol, 1 equiv), Pd(PPh3)4 (5 mol%), dry THF (4 mL), N2 atmosphere; K2CO3 (6 equiv), water (1 mL); boronic acid (1.2 equiv), dry THF (1 mL); 70 °C, overnight. ^b^ Isolated yields.

**Table 4:** Absorption maximum of the first electronic transition (λ_abs, max_), emission maximum (λ_em, max_), Stokes shift, full width at half maximum of the emission (FWHM_em_), and fluorescence quantum yield (*φ*) of the investigated compounds in solution.

	TAP	λ_abs, max_ (nm)	λ_em, max_ (nm)	Stokes Shift (cm^−1^)	FWHM_em_ (cm^−1^)	*φ* (%)
	**1** [3]	399	476	4050 ± 50		79
DCM (RT)	**4a**	424	503	3700 ± 50	3420 ± 50	12
	**4b**	428	523	4240 ± 50	3610 ± 50	28
	**4c**	441	476	1670 ± 50	2570 ± 50	1
	**4e**	430	525	4210 ± 50	3660 ± 50	27
	**5a**	427	529	4520 ± 50	3730 ± 50	14
ACN (RT)	**7**	394	512	5850 ± 50	4950 ± 50	2
	**8**	418	522	4770 ± 50	4020 ± 50	4
MeTHF (77 K)	**4a**	439	457	900 ± 50	2290 ± 50	
	**4b**	452	466	660 ± 50	2190 ± 50	
	**4c**	453	462	430 ± 50	1770 ± 50	
	**4e**	452	466	660 ± 50	2090 ± 50	
	**5a**	445	467	1060 ± 50	2330 ± 50	
	**7**	409	423	810 ± 50	2200 ± 50	
	**8**	433	445	620 ± 50	2050 ± 50	

## Data Availability

The original contributions presented in the study are included in the article/Supplementary Material; further inquiries can be directed to the corresponding authors.

## Supplementary Materials

The following supporting information can be downloaded at: https://www.mdpi.com/article/10.3390/molecules29102229/s1, Figures S1–S17: NMR spectra; Figure S18: Crystal structure of **TAP4a**.
