# Supplementary material for: Palladium-Catalyzed Arylations towards 3,6-Diaryl-1,3a,6a-triazapentalenes and Evaluation of Their Fluorescence Properties
Source: Molecules. 2024 May 9;29(10):2229. doi: 10.3390/molecules29102229 (PMC11123795; doi:10.3390/molecules29102229)
Supplement: Supplementary file 1 [file molecules-29-02229-s001.zip › molecules-2983333-supplementary.pdf]

Palladium-Catalyzed Arylations Towards 3,6-Diaryl-1,3a,6a-triazapentalenes and Evaluation of Their Fluorescence Properties

Yingchun Wang,[a] Tomas Opsomer,*[a] Flip de Jong,[b] Davy Verhaeghe,[b] Maarten Mulier,[a] Luc Van Meervelt,[c] Mark Van der Auweraer,[b] Wim Dehaen*[a]

[a]Sustainable Chemistry for Metals and Molecules, Department of Chemistry, KU Leuven, Celestijnenlaan 200F, 3001 Leuven, Belgium.

[b]Molecular Imaging and Photonics, Department of Chemistry, KU Leuven, Celestijnenlaan 200F, 3001 Leuven, Belgium.

[c]Biochemistry, Molecular and Structural Biology, Department of Chemistry, KU Leuven, Celestijnenlaan 200F, 3001 Leuven, Belgium

*Email: tomas.opsomer@sckcen.be, wim.dehaen@kuleuven.be

Contents

1D NMR spectra 2

2D NMR spectra 10

Crystal structure of **TAP4a** 12

1D NMR spectra

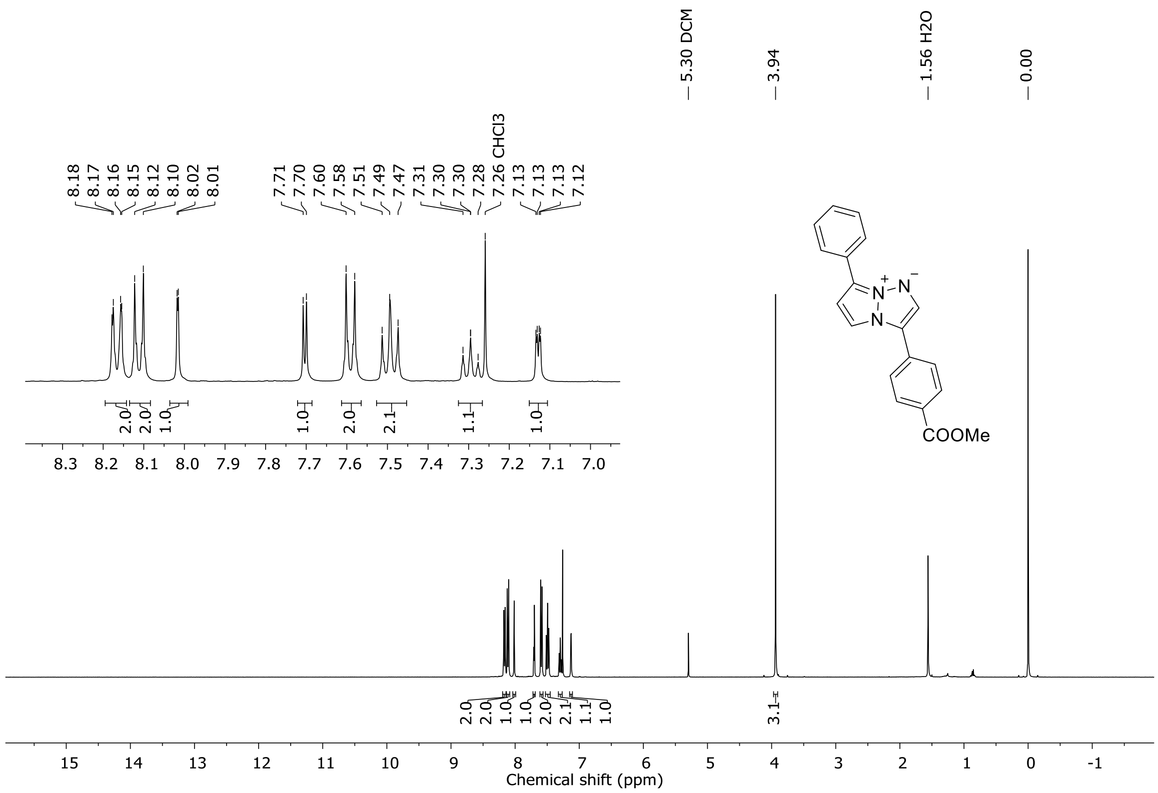

Figure S1. **TAP4a,** ^{1}H, 400 Hz, $CDCl_3$

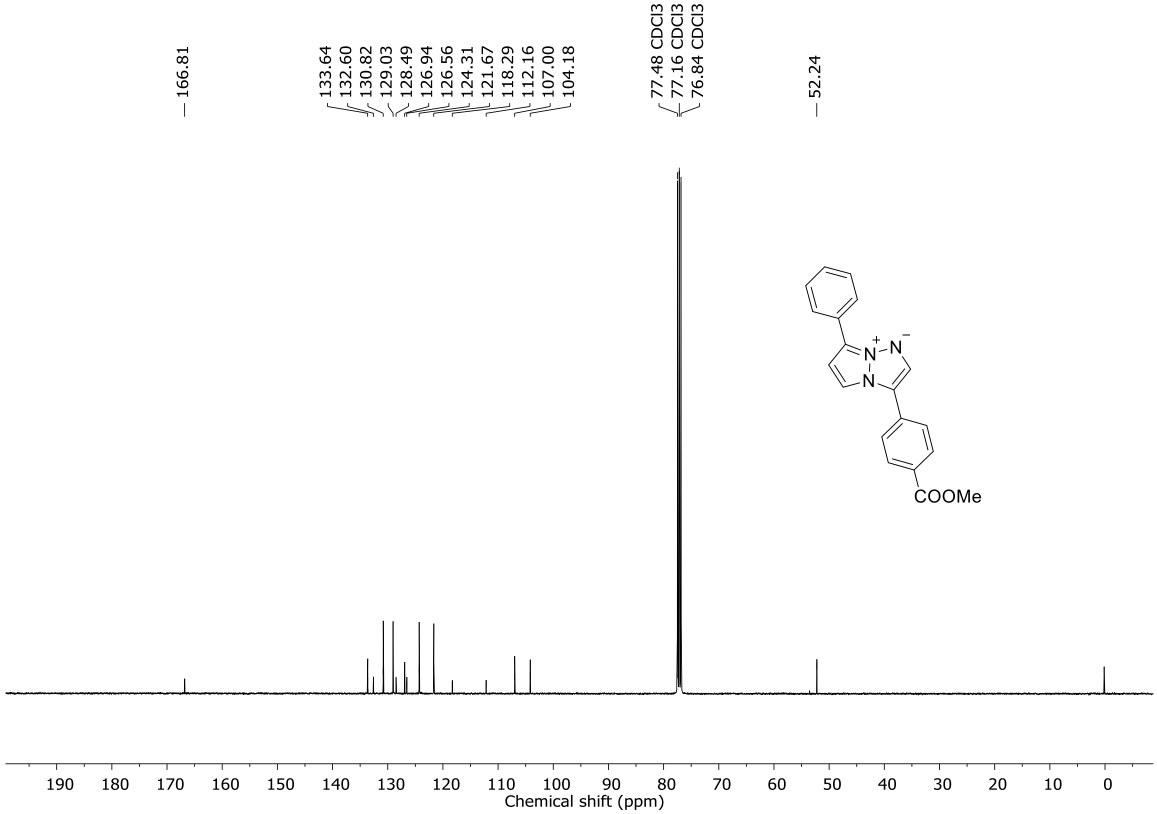

Figure S2. **TAP4a,** $^{13}C\{^{1}H\}$, 101 MHz, $CDCl_3$

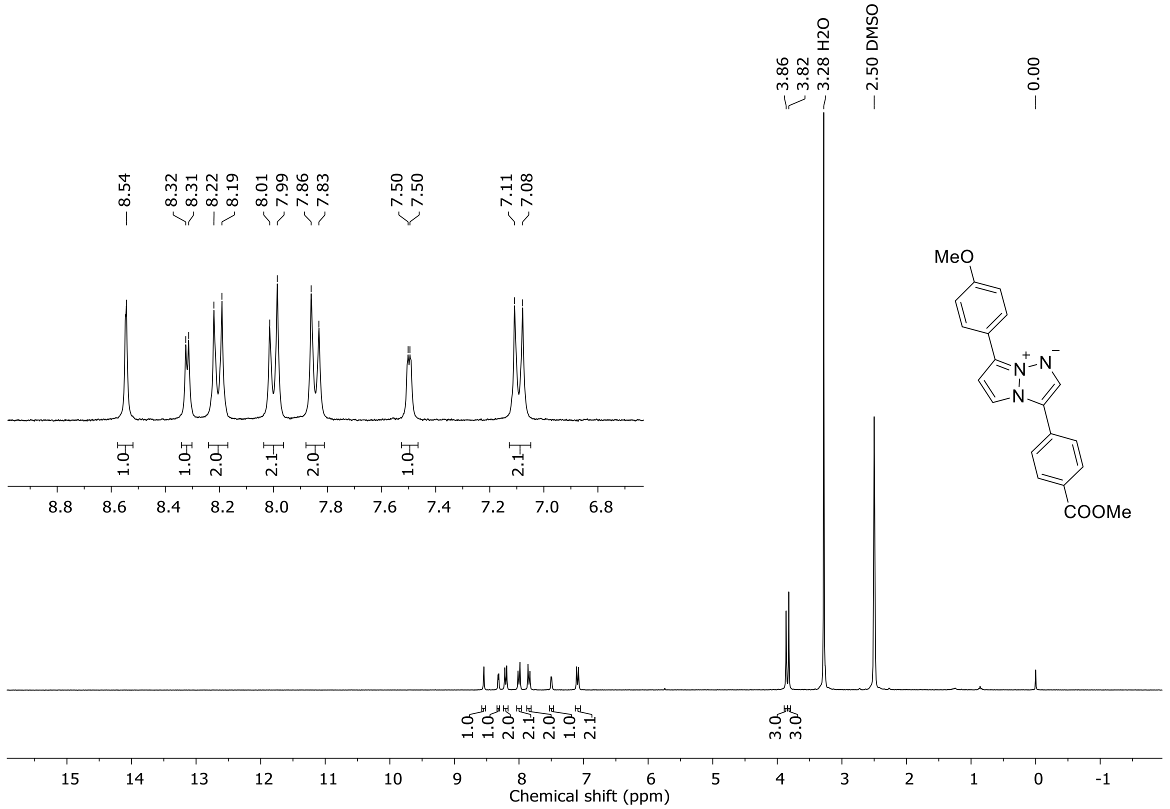

Figure S3. **TAP4b,** ^{1}H, 300 Hz, DMSO-d_6

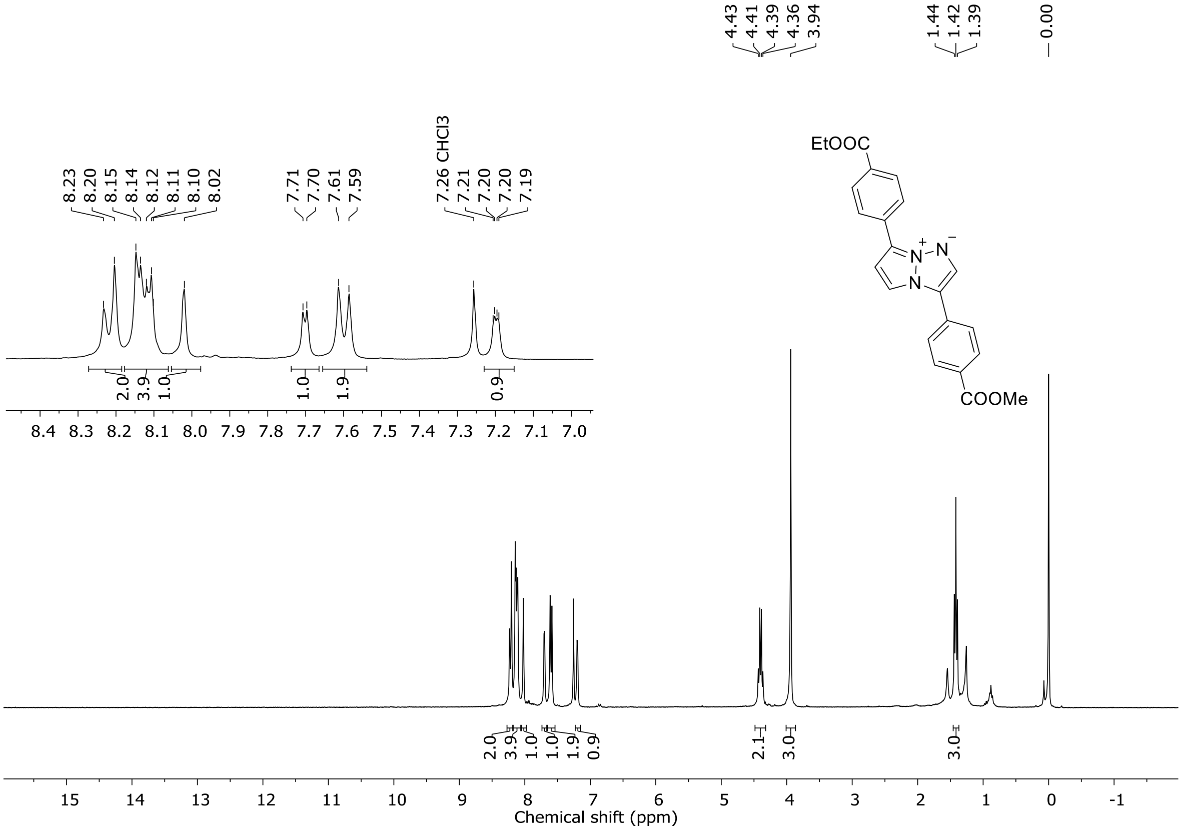

Figure S4. **TAP4c,** ^{1}H, 300 MHz, $CDCl_3$

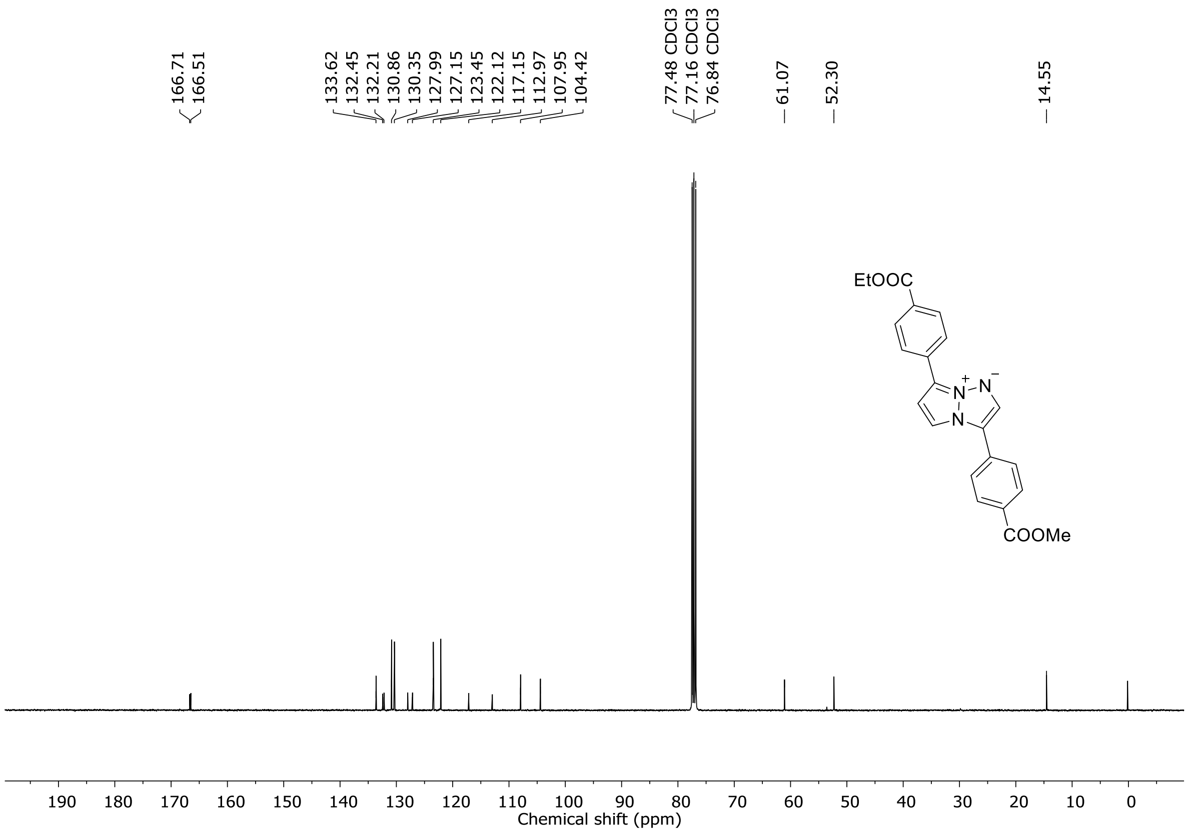

Figure S5. **TAP4c,** $^{13}C\{^{1}H\}$, 101 MHz, $CDCl_3$

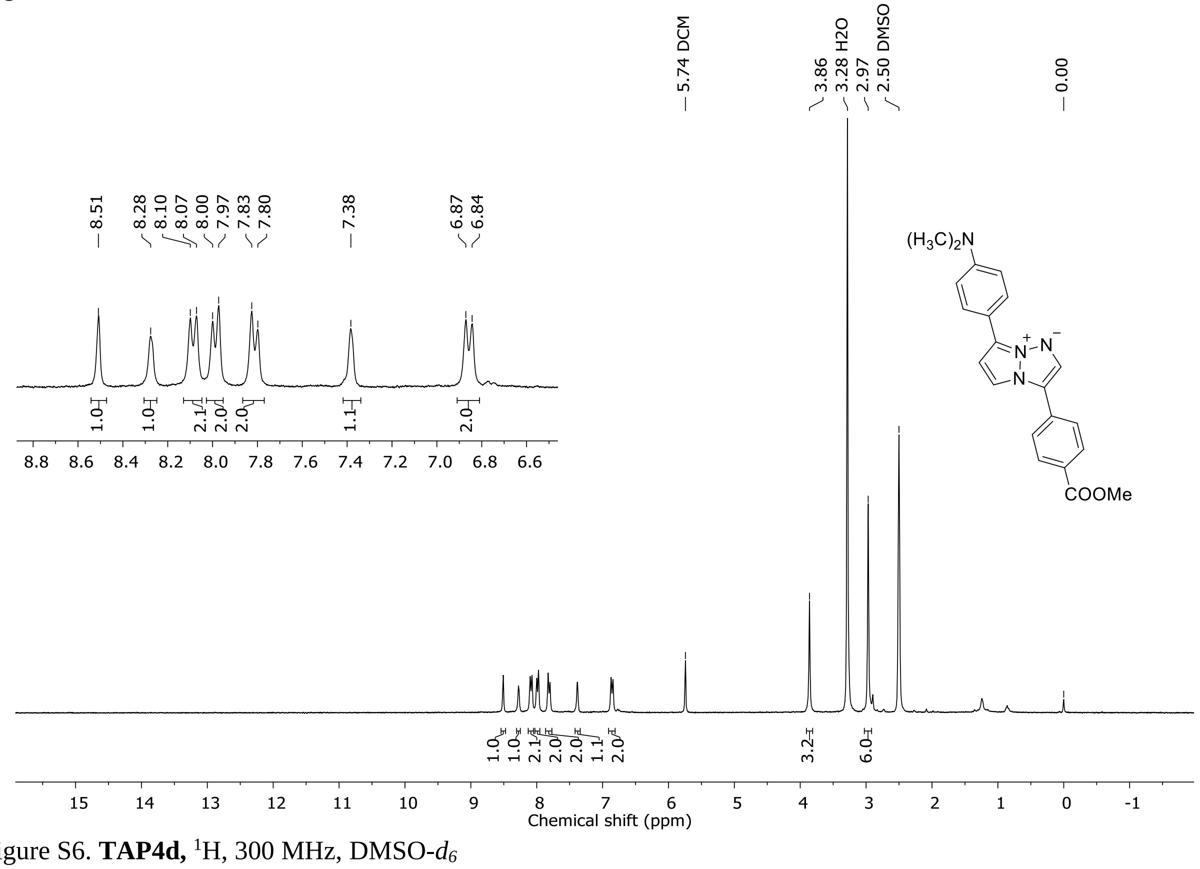

Figure S6. **TAP4d,** ^{1}H, 300 MHz, DMSO-d_6

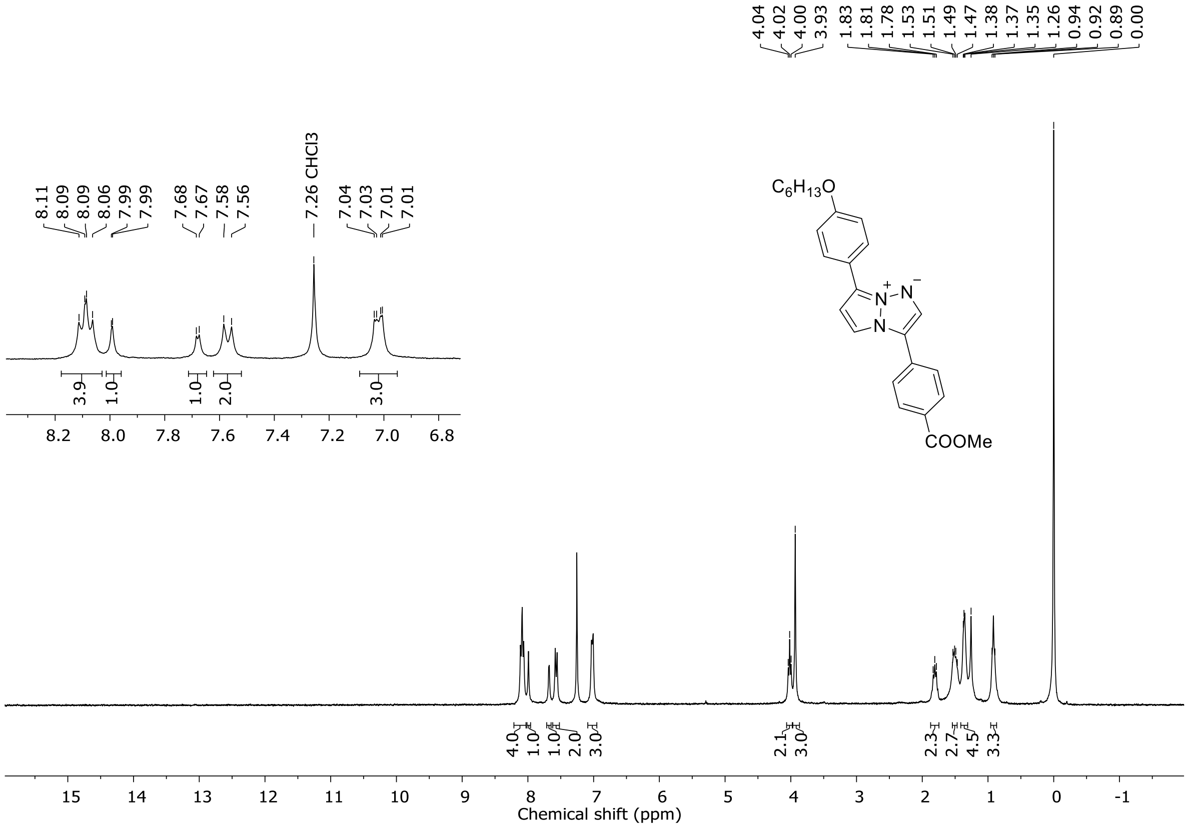

Figure S7. **TAP4e,** ^{1}H, 300 MHz, $CDCl_3$

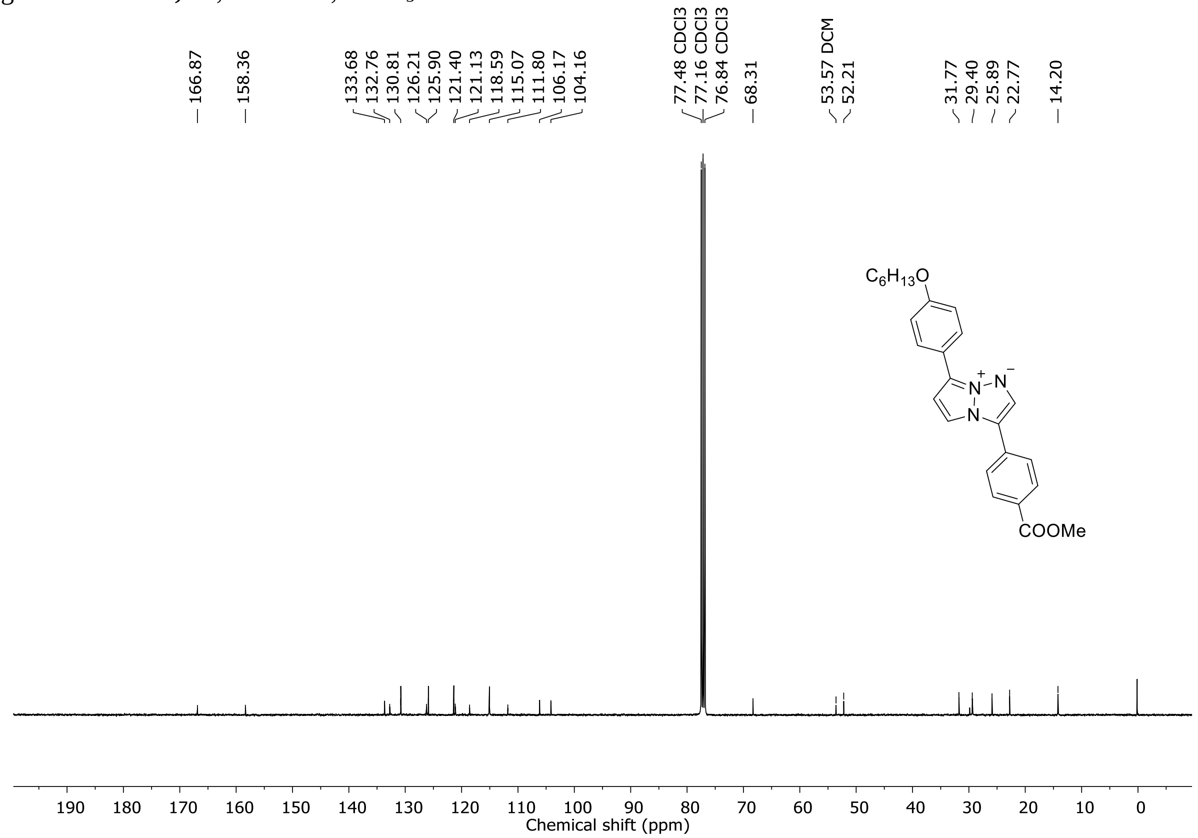

Figure S8. **TAP4e,** $^{13}C\{^{1}H\}$, 101 MHz, $CDCl_3$

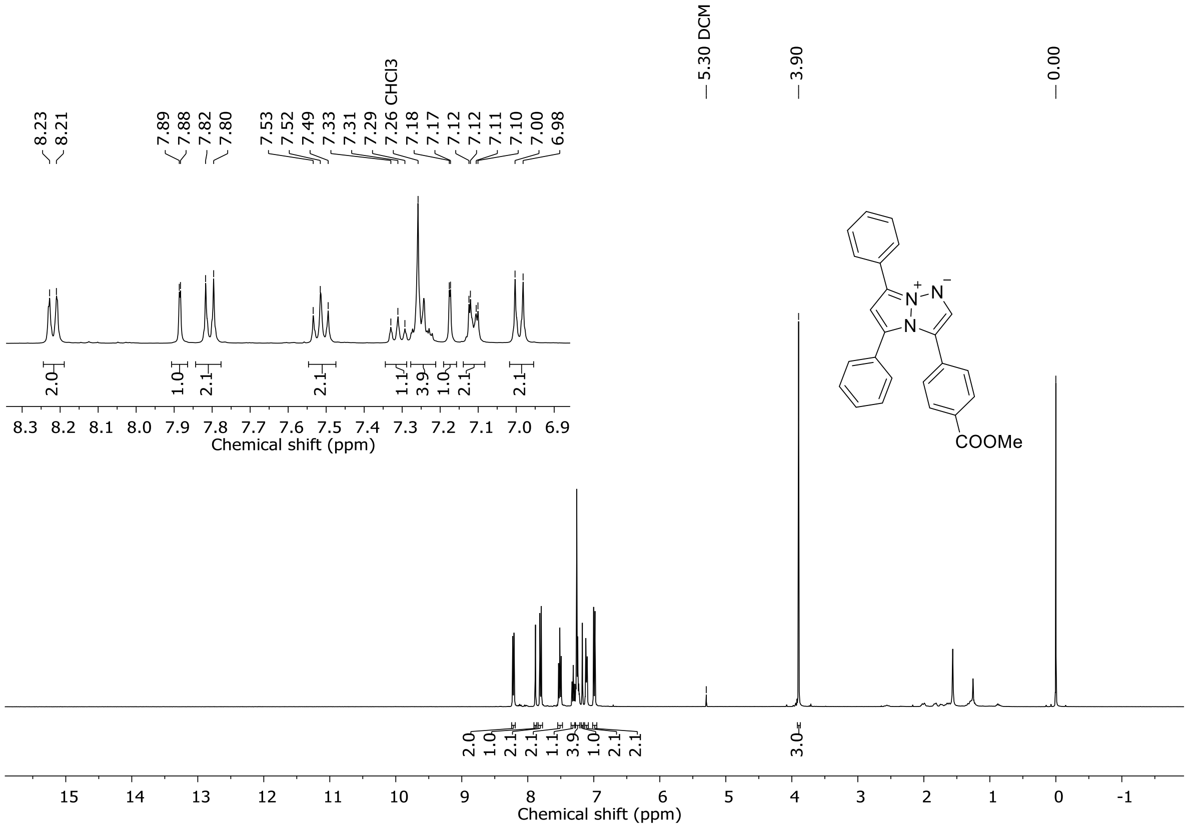

Figure S9. **TAP5a**, ^{1}H, 400 MHz, $CDCl_3$

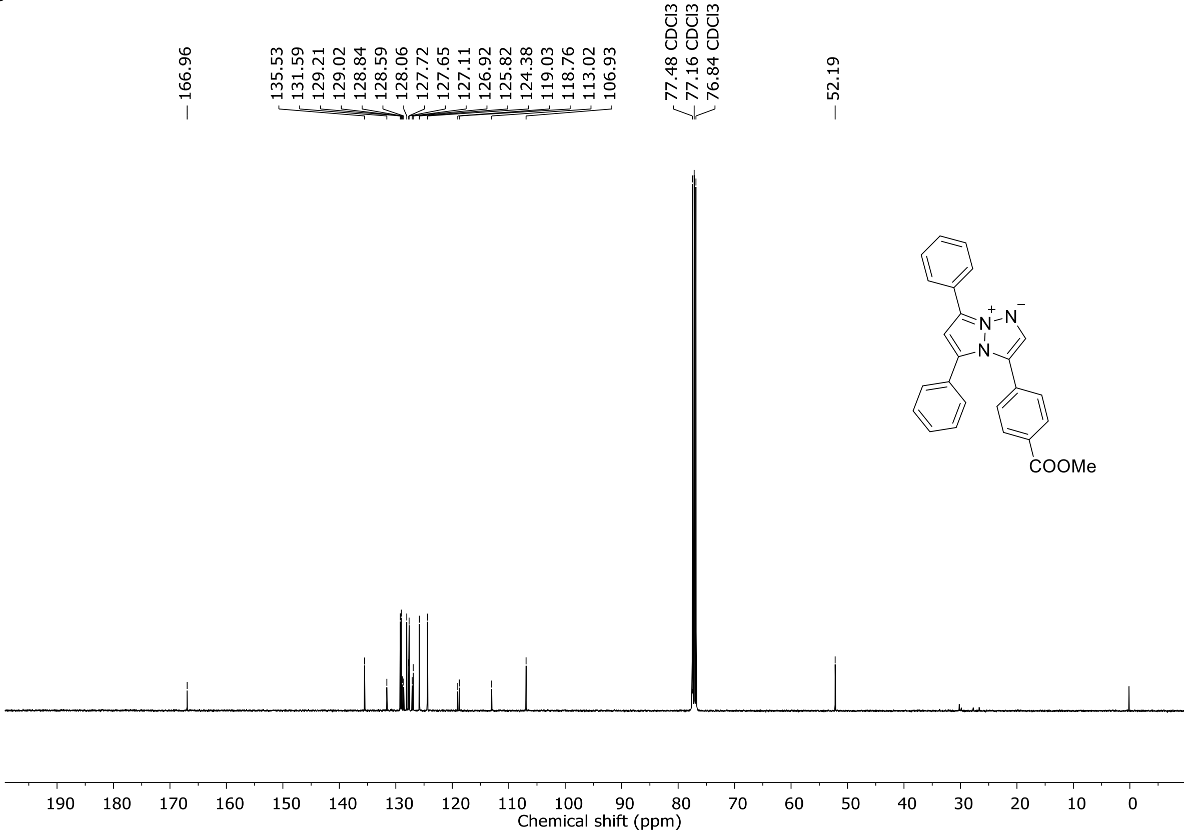

Figure S10. **TAP5a,** $^{13}C\{^{1}H\}$, 101 MHz, $CDCl_3$

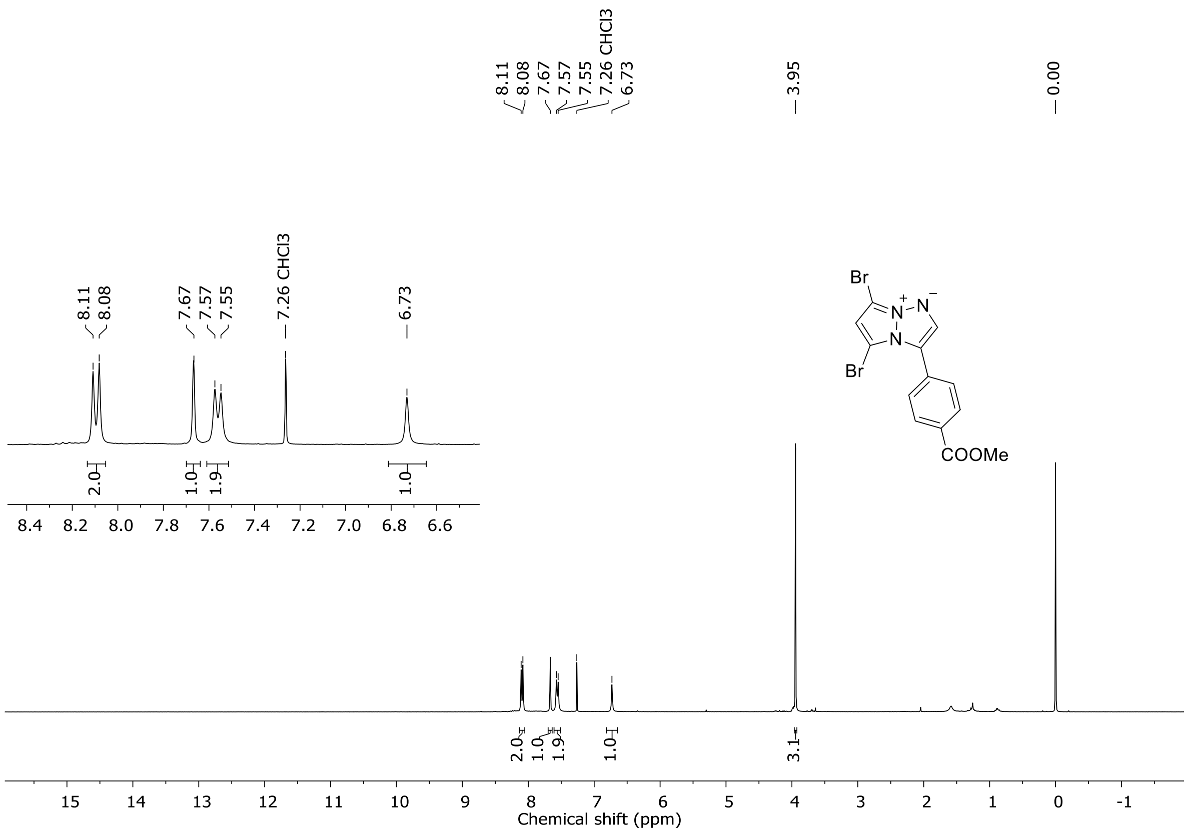

Figure S11. **TAP6b**, ^{1}H, 400 MHz, $CDCl_3$

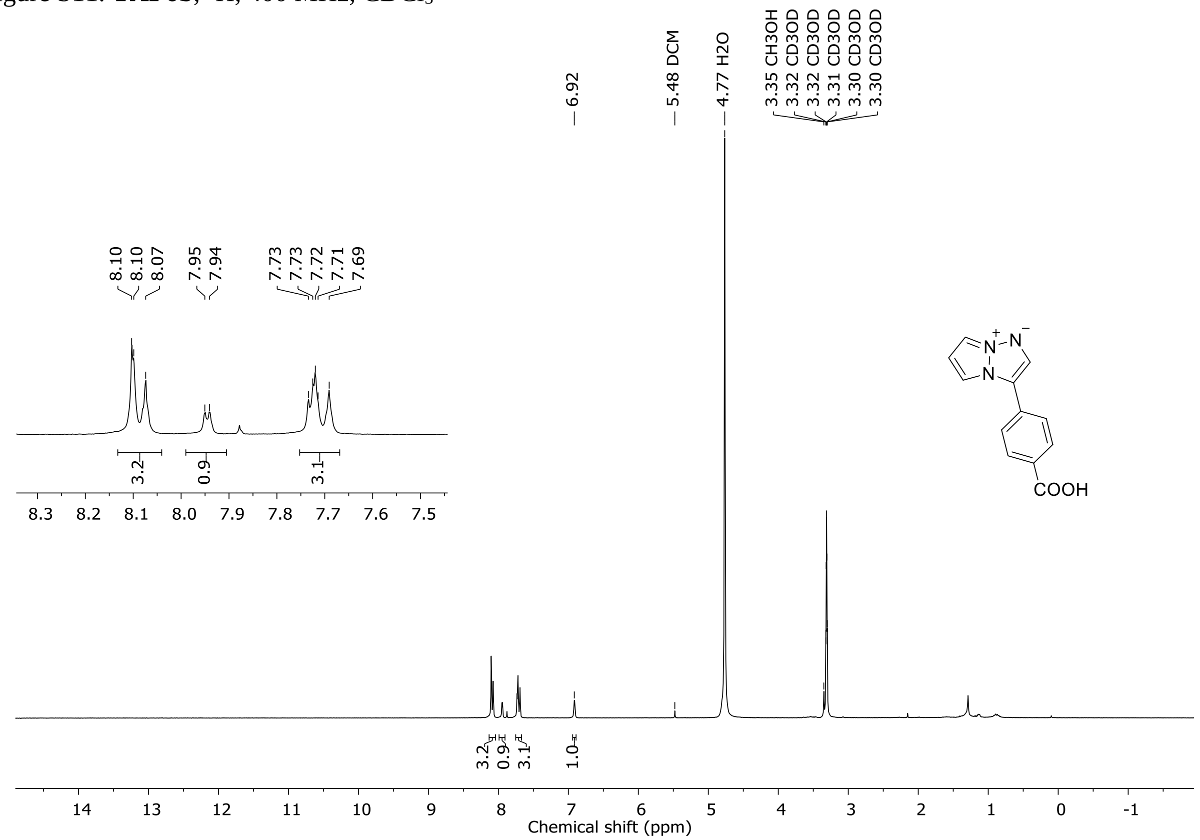

Figure S12. **TAP7**, ^{1}H, 300 MHz, CD_3OD

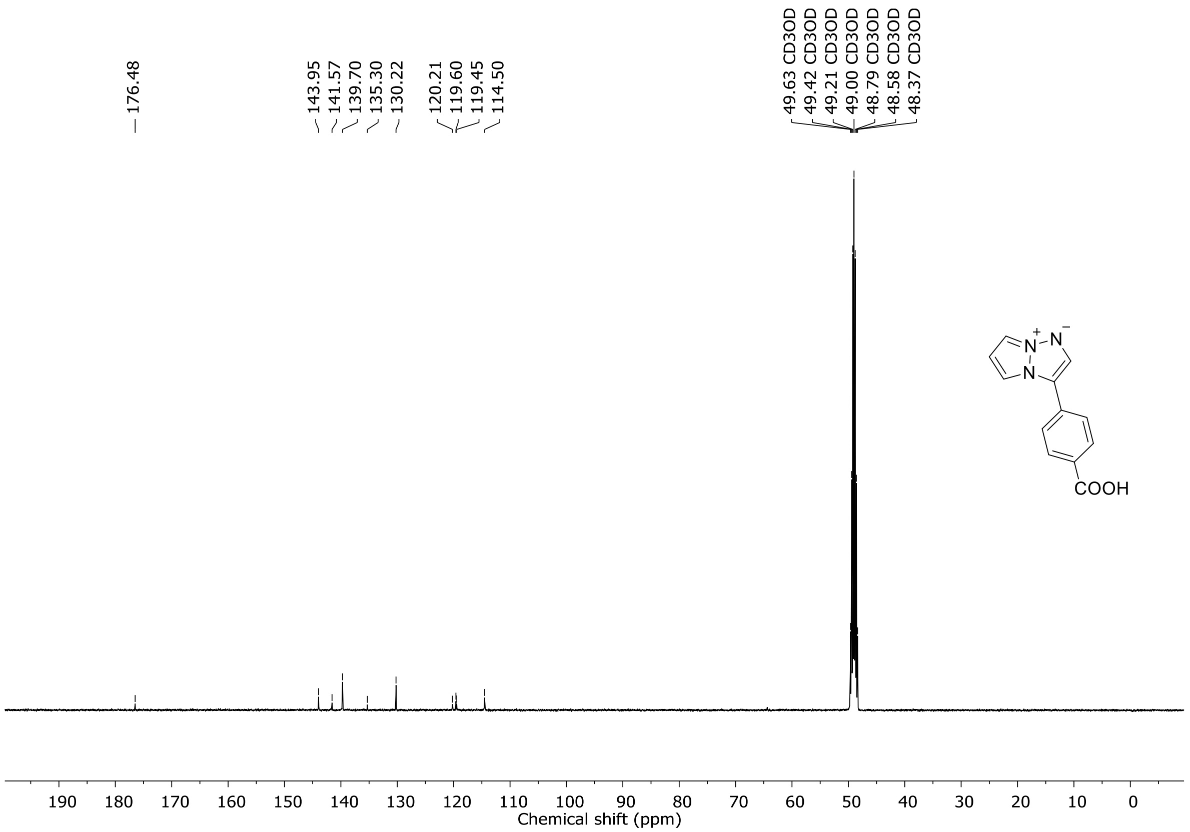

Figure S13. **TAP7,** $^{13}C\{^{1}H\}$, 101 MHz, CD_3OD

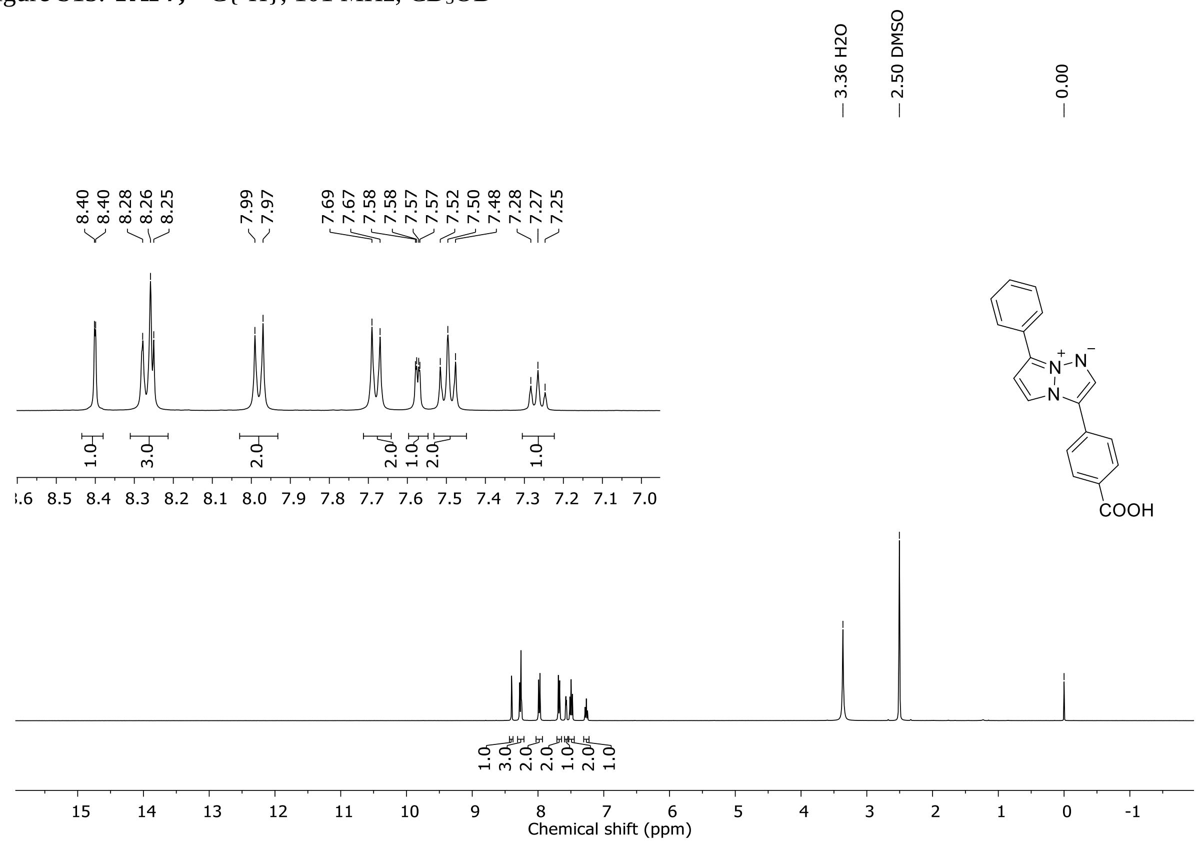

Figure S14. **TAP8,** ^{1}H, 400 MHz, DMSO-d_6

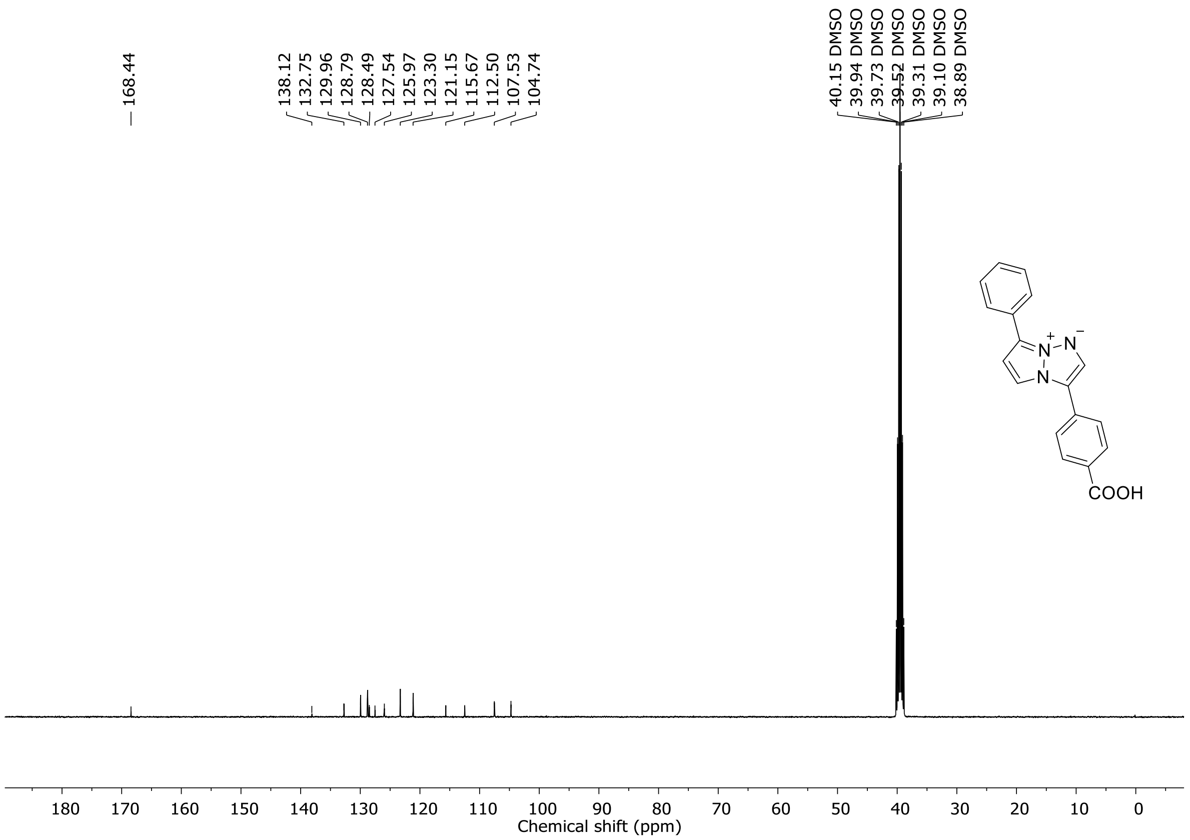

Figure S15. **TAP8,** $^{13}C\{^{1}H\}$, 101 MHz, DMSO-d_6

2D NMR spectra

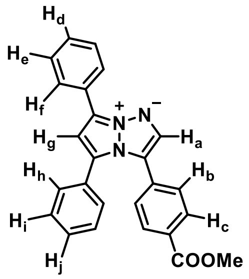

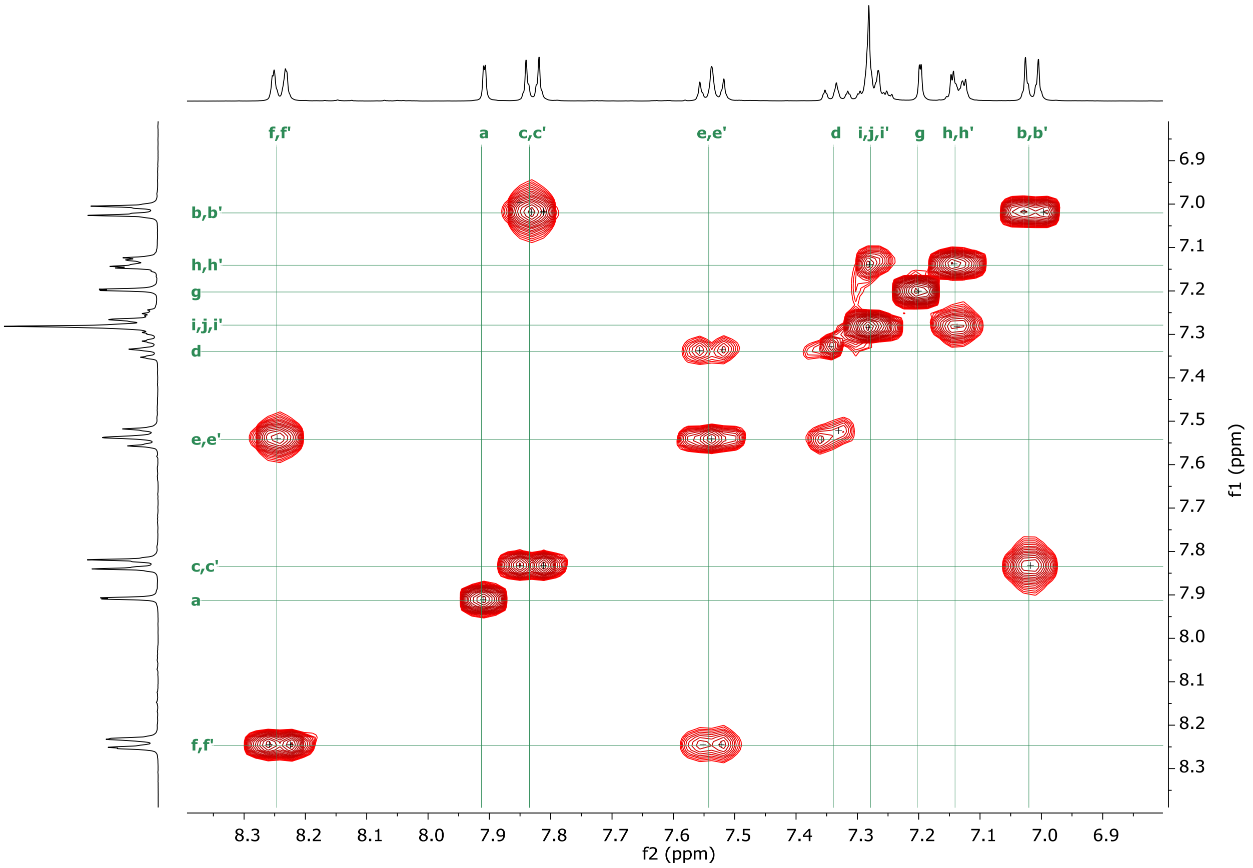

Figure S16. Structure and ^{1}H-^{1}H COSY NMR spectrum (400 MHz, $CDCl_3$) of **TAP5a**.

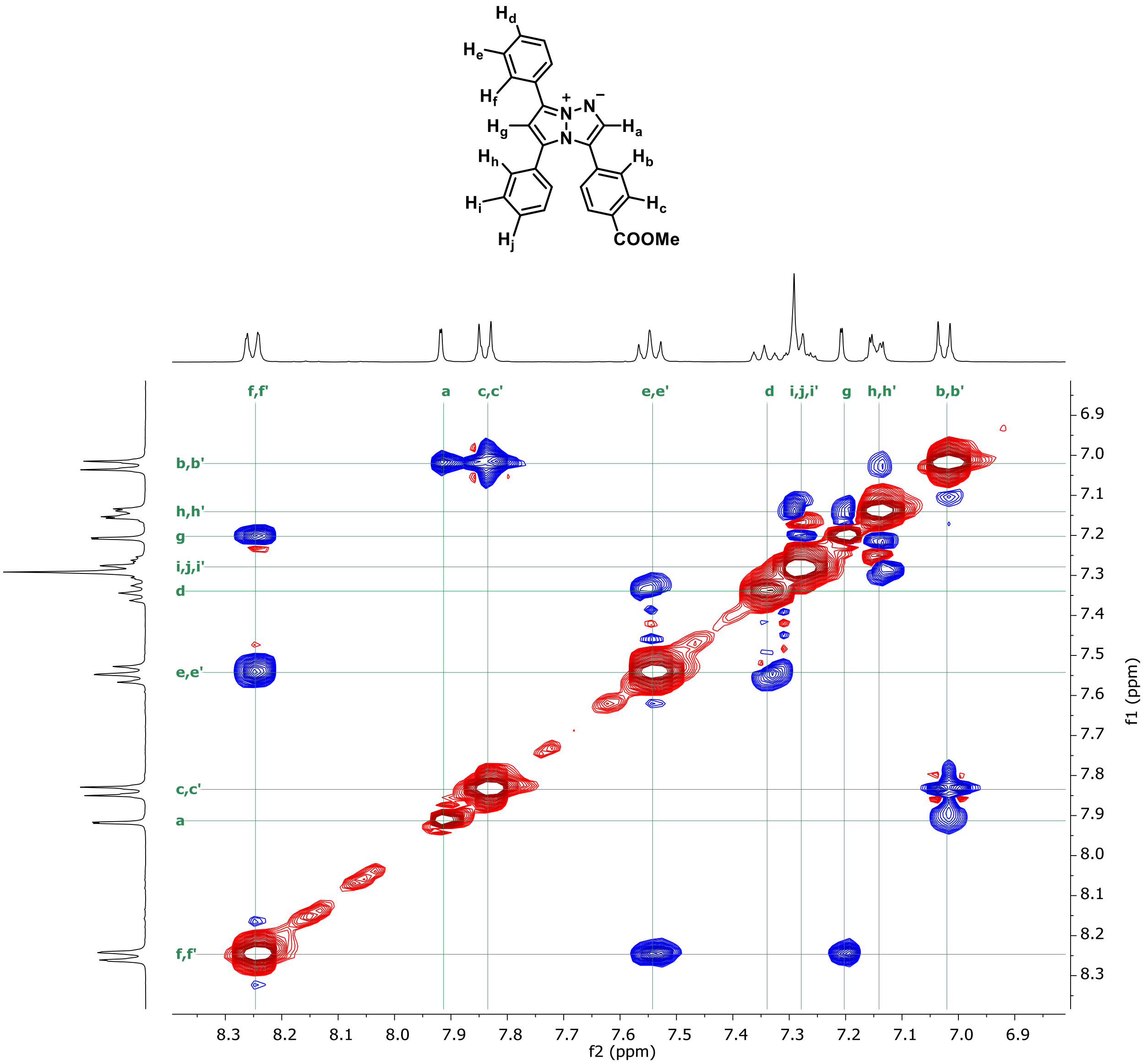

Figure S17. Structure and ^{1}H-^{1}H NOESY NMR spectrum (400 MHz, $CDCl_3$) of **TAP5a**.

Crystal structure of TAP4a

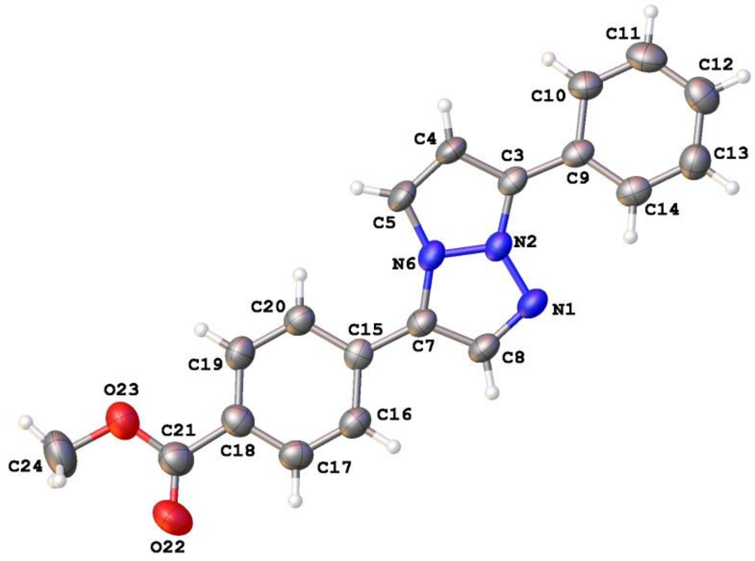

Figure S18. Crystal structure of **TAP4a**. Thermal ellipsoids are drawn at the 50% probability level.
